# Clinical and Epidemiological Profiles of Primary Healthcare Professionals with COVID-19 Infection and Long COVID: An Observational Study

**DOI:** 10.3390/healthcare11121677

**Published:** 2023-06-07

**Authors:** Esperanza Romero-Rodríguez, Rodrigo Vélez-Santamaría, Luis Ángel Pérula-de-Torres, Jesús González-Lama, Rafael Ángel Castro-Jiménez, Lucía Simón-Vicente, Celia Jiménez-García, Jerónimo J. González-Bernal, Mirian Santamaría-Peláez, Jessica Fernández-Solana, Josefa González-Santos

**Affiliations:** 1 Maimonides Institute for Biomedical Research of Córdoba (IMIBIC), Reina Sofía University Hospital, Córdoba University, 14004 Córdoba, Spain; luisangel.perula@gmail.com (L.Á.P.-d.-T.); jegonla@telefonica.net (J.G.-L.); rangel.castro.sspa@xn--juntadeandalucia-ipb.es (R.Á.C.-J.); celia.jimenez.sspa@xn--juntadeandalucia-ipb.es (C.J.-G.); 2 Córdoba and Guadalquivir Health District, 14011 Córdoba, Spain; 3 Carlos Castilla del Pino Clinical Management Unit, 14011 Córdoba, Spain; 4 Department of Health Sciences, University of Burgos, 09001 Burgos, Spain; rvs0014@alu.ubu.es (R.V.-S.); lsvicente@ubu.es (L.S.-V.); jejavier@ubu.es (J.J.G.-B.); mspelaez@ubu.es (M.S.-P.); mjgonzalez@ubu.es (J.G.-S.); 5 Cabra Clinical Management Unit, “Matrona Antonia Mesa Fernández” Health Center, AGS South of Córdoba, 14940 Córdoba, Spain; 6 Especialista en Medicina Familiary Comunitaria, Reina Sofía University Hospital, 14004 Córdoba, Spain

**Keywords:** long COVID, persistent COVID, post COVID-19, COVID-19, symptoms, pathology, health professional

## Abstract

Health professionals have been one of the groups most affected by the SARS-CoV-2 virus. Currently, there is little scientific evidence on the similarities and differences between COVID-19 infection and the development of long COVID in primary care (PC) workers. Therefore, it is necessary to analyse their clinical and epidemiological profiles in depth. This study was observational and descriptive, including PC professionals who were divided into three comparison groups based on the diagnostic test for acute SARS-CoV-2 infection. The responses were analysed using descriptive and bivariate analysis to examinate the relationship between independent variables and the presence or not of long COVID. Binary logistic regression analysis was also conducted, with each symptom as the dependent variable and each group as the independent variable. The results describe the sociodemographic characteristics of these population groups, revealing that women in the health sector are the most affected by long COVID and that being in this group is associated with its development. Furthermore, individuals with long COVID exhibited the highest number of symptoms and pathologies. Certain symptoms were found to be associated with long COVID development in this population, including an altered sense of smell, pneumonia, fever, and sore throat, among others. Similarly, altered senses of smell and taste, chest tightness, and joint pain, among others, were found to be associated with acute COVID-19 infection. Additionally, patients with pre-existing overweight or obesity were more likely to experience acute COVID-19 and develop long COVID. The data obtained can be crucial for improving the detection, diagnosis, and treatment of long COVID patients, ultimately leading to an enhancement in their quality of life.

## 1. Introduction

The symptomatology associated with COVID-19 is a topic of public health interest due to its rapid transmission and the emergence of new virus variants on all continents. The appearance of these new variants poses an even greater challenge to public health, representing a significant danger to the total control of the pandemic [1,2]. While their definitive origin is still unknown, the most probable theory, according to the World Health Organization (WHO), is that of zoonotic origin [3]. The rapid transmission of the virus occurs through the inhalation of respiratory droplets or aerosols emitted by an infected person [4,5].

COVID-19 infection can manifest as asymptomatic or with mild, moderate or severe symptoms. The range of symptoms associated with the infection is quite broad and can affect various organs and systems [6]. The clinical features of COVID-19 have been extensively studied in both Spanish [7,8] and international studies [9,10,11]. The most common symptoms include fever, dry cough, tiredness or fatigue, dyspnoea, pharyngeal pain, chills and diarrhoea. These symptoms described during the acute phase of the disease can persist over time, leading to persistent COVID-19 or long COVID [12,13].

The absence of a standardized and globally agreed-upon definition makes it challenging to categorize the epidemiology of long COVID and develop potential treatments. To address this issue, the WHO has defined post-COVID condition as an illness that occurs in individuals with a history of probable or confirmed SARS-CoV-2 infection, typically occurring 3 months after the onset of symptoms and lasting for at least 2 months, with symptoms that cannot be explained by an alternative diagnosis [14].

The Spanish Society of General Medicine conducted a survey describing the general characteristics of patients with persistent COVID-19 diagnosed during the first wave of the pandemic in the first year [15]. The survey revealed factors such as age (50% of participants aged 36–50 years), sex (more frequent in women), and common symptoms (asthenia, malaise, headache, or mood disturbance) associated with persistent COVID-19 symptoms. The estimated prevalence of persistent COVID-19 in this study was approximately 15% and was increasing. During the early stage of the pandemic, a significant portion of the population faced changes in accessing diagnostic tests, and there was a lack of research on population groups at higher risk of virus exposure.

Many aspects of persistent COVID are still poorly understood, and some studies suggest that this phenomenon may actually encompass multiple different syndromes [16,17]. Specifically, it is unknown which individuals will fully recover and which will continue to experience persistent symptoms [16]. Identifying high-risk individuals would enable the development of strategies to mitigate or prevent symptom persistence [18,19].

It is worth mentioning that vaccination has proven to be an important preventive strategy for long COVID, as it is highly effective and efficient in preventing severe infection and hospitalizations due to COVID-19 [20,21]. Additionally, there are medications available for severe forms of COVID-19 that help reduce the risk of hospitalization or death among high-risk individuals [22]. However, the Ministry of Health in Spain concluded that the potential benefits of vaccination or these medications on the symptoms associated with long COVID are still unclear, although they do not appear to worsen the course of the disease [21].

Due to the mechanism of virus transmission, healthcare professionals are particularly susceptible to infection and transmission. A study utilizing data from the UK Biobank [23] investigated the differences in severe COVID-19 manifestations among various worker groups. The data revealed that healthcare professionals, education and social workers, and other essential workers were at higher risk of developing severe COVID-19 compared to non-essential workers. An international review of coronavirus infection epidemiology and risk factors among healthcare workers in 2020 indicated that healthcare professionals represented a significant proportion of individuals infected with the coronavirus, with a particularly high incidence of infection following unprotected exposures [24].

Preliminary results of the “Factors of COVID-19 Diffusion” project in Spain have demonstrated that the percentage of infected healthcare professionals was a relevant factor contributing to the spread of the pandemic during its upswing [25]. Data from the Spanish seroprevalence study show a higher prevalence of antibodies among health workers compared to other population groups [26], accounting for approximately 10% of cases. Currently, there is a lack of research specifically explaining persistent COVID-19 among primary care (PC) professionals in Spain, who provide initial care to COVID-19 patients. It is important to better understand the factors affecting this population in order to guide public health measures, protect healthcare workers and their families, maintain a functioning healthcare system, and control secondary transmission rates within the community [27,28,29].

Given that healthcare professionals have been one of the most affected groups, the aim of this study was to conduct a more comprehensive analysis of their clinical and epidemiological profiles. Additionally, it was important to describe the clinical and epidemiological profiles of patients with long COVID treated in PC and compare them with the profiles of patients with acute COVID-19 infection and those without COVID-19 infection. On the other hand, it was also considered important to study the symptoms that turned out to be associated with a higher prevalence of being infected with COVID-19 or developing long COVID when experiencing them.

## 2. Materials and Methods

### 2.1. Study Design

We conducted an observational, descriptive, multicentric study with three comparison groups, classified according to the results of the acute infection diagnostic test (ADT: antigen detection test and/or polymerase chain reaction (PCR)), by SARS-CoV-2, and the existence or not of long COVID-19 or post-COVID-19 syndrome, following the WHO definition [30].

The groups were: (1) “Cases”: patients with acute infection by SARS-CoV-2 (positive ADT) and long COVID; (2) “Controls 1”: patients with acute infection by SARS-CoV-2 (positive ADT) and absence of criteria for long COVID; (3) “Controls 2”: individuals without acute infection by SARS-CoV-2, for whom an ADT had been requested for presenting an acute clinical picture due to the presence of COVID-19 or for being a close contact of a confirmed case of COVID-19.

### 2.2. Study Population

The study included PC professionals of the Spanish National Health System (NHS).

Selection criteria were as follows: (1) being a health professional of the NHS; (2) meeting clinical or epidemiological criteria for suspected COVID-19 disease and/or long COVID (Suspected COVID-19 disease was defined as the presence of acute respiratory symptoms consistent with sudden onset in the last 10 days, including cough, dyspnoea, sore throat or rhinitis, with or without fever. Other symptoms such as anosmia, ageusia, diarrhoea, chest pain or headache, among others, were also considered symptoms of suspected SARS-CoV-2 infection. From the epidemiological perspective, suspected COVID-19 disease was also considered if the healthcare professional had been in close proximity, less than 2 m, to a confirmed case for a cumulative time of more than 15 min in a 24 h period without adequate protection measures. Long COVID was defined as a symptomatic condition that persisted in patients who had had COVID-19 and continued to experience symptoms after the acute phase of the disease, lasting for at least 4 weeks from the onset of symptoms [14,30]); and (3) undergoing an ADT and consenting to participate in the study. In the questionnaire, patients indicated whether the diagnosis of COVID was made by antigen test or real-time polymerase chain reaction (RT-PCR, on a nasal/pharyngeal swab). Exclusion criteria was as follows: refusal to participate in the research study.

The Office for National Statistics of the United Kingdom has estimated that one in 10 patients diagnosed with COVID-19 have symptoms beyond 12 weeks after diagnosis and, therefore, a long COVID prevalence of 10% was assumed [31]. Therefore, a sample of 138 individuals would be sufficient to achieve the objective proposed for this study, for a confidence level of 95% and a precision of ±5 percentage points (calculations made with the Granmo programme: https://www.imim.es/ofertadeserveis/software-public/granmo/ (accessed on 13 February 2021)).

### 2.3. Procedure

The enrolment procedure was initiated to deepen the clinical-epidemiological analysis of patients with persistent COVID-19, which led to the initiation of the EPICOVID-AP21 investigation. The information from study participants was obtained through an online questionnaire created using the Google Forms tool. The questionnaire was sent to members of the Spanish Society of Family and Community Medicine and the Spanish Society of General Practitioners. Each participant received an invitation and a link to access the online questionnaire. Interested participants completed the questionnaire online before providing informed consent.

The research project received authorization from the management of the Cordoba and Guadalquivir Health District and the Cordoba South Health Management Area. Additionally, it obtained approval from the Clinical Research Ethics Committee of the Reina Sofia Hospital in Cordoba (Ethical certificate number: 5033). Informed consent was obtained from the patients participating in the study, ensuring voluntariness. Data processing was conducted in compliance with the European Data Protection Regulation and Organic Law 3/2018 on Personal Data Protection and guarantee of digital rights.

### 2.4. Statistical Analysis

The questionnaire data were stored in a survey created with Google Forms in Google Drive. These data were exported to an Excel spreadsheet and analysed with the statistical software SPSS version 17.0 (IBM Inc., Chicago, IL, USA). A descriptive and comparative analysis of the groups was conducted. Confidence intervals of 95% (95% CIs) were estimated for the main parameters of the study.

Bivariate analysis was performed to determine whether there was any relationship between groups (healthcare professionals and general population) and to test the relationship between the independent variables and the presence or absence of persistent COVID-19 disease. For this analysis, the Chi-square test was used, as well as mean comparison test for independent samples such as Student’s *t*-test or ANOVA (after testing normality using the Kolmogorov–Smirnov test). If the data did not meet the assumption of normality, non-parametric tests such as the Kruskal–Wallis test were used. Bilateral contrasts were used, and a significance level of *p* ≤ 0.05 was applied.

A binary logistic regression analysis was performed to test which variables were associated with the prevalence of having acute COVID-19 and long COVID. The dependent variables were: acute COVID-19 (yes vs. no) and long COVID (yes vs. no). The independent variables included in the regression model were: sociodemographic variables (age—treated as a dummy variable, sex, location, and clinical pathologies (only those pathologies with a frequency of >10 were included in the model)). Odds ratio (OR) values and their respective 95% confidence intervals (95% CIs) were found for each variable. Statistical analysis was performed using the IBM Statistic SPSS 20 program with a significance level of 95% (*p* = 0.05).

## 3. Results

Of the 1490 primary care professionals included in the study, 61.6% were doctors, 8.4% were nurses, 6% were nursing assistants, and the rest belonged to other categories such as physiotherapists, occupational therapists, speech therapists, nutritionists, pharmacists, psychologists, etc. Furthermore, 74.9% lived in urban areas (>20,000 inhabitants), and 25.1% lived in rural areas, with no significant differences between comparison groups.

Regarding COVID status, 73% of the participants did not have COVID-19 (negative ADT results), 16.8% had acute COVID infection, and 10.2% of the patients had the clinical picture of long COVID. The patients with long COVID had a mean duration of 35.23 ± 20.70 weeks (95% CI: 31.89–38.57; range = 8–114 weeks). The mean age of the entire sample was 46 ± 11.37 years (95% CI: 46.53–47; range = 14–94 years), with no statistically significant differences between the comparison groups (Table 1). Significant differences were observed in the diagnosis of long COVID based on sex, with 84.9% (CI 95%: 79.2–90.6%) identified in women and 15.1% (CI 95%: 14.6–27.5) identified in men (*p* = 0.007).

Of the patients in the study, 81.2% were symptomatic. The mean number of clinical signs or symptoms experienced by the patients was 4.44 ± 6.05 (95% CI: 4.18–4.74; median = 2; range: 0–35). Significant differences were observed by group, with an average number of symptoms in patients without COVID-19 of 2.64 ± 4 (95% CI: 2.42–2.86; range = 0–24), in patients with acute COVID-19 infection of 6.38 ± 5.26 (95% CI: 5.81–6.96; range = 0–24), and in those with long COVID of 14.60 ± 8.78 (CI 95%: 13.25–15.94; range = 1–35) (Kruskal–Wallis; *p* < 0.001).

Table 2 shows the results of the binary regression model considering sociodemographic characteristics of patients with acute COVID-19 and long COVID. In those with long COVID, significant results were obtained according to sex (higher prevalence of long COVID in women; OR: 1.90; 95% CI: 1.32–2.74; *p* = 0.001).

Table 3 present the results for the symptoms, signs and clinical presentation studied, categorized according to the comparison group. Patients with long COVID-19 exhibited a higher prevalence of fatigue (78.9%; *p* < 0.001), headache (69.7%; *p* < 0.001), muscle or joint pain (63.2%; *p* < 0.001) and dyspnoea (61.2%; *p* < 0.010) compared to those with acute COVID-19 or individuals without COVID-19.

**Figure 1:**
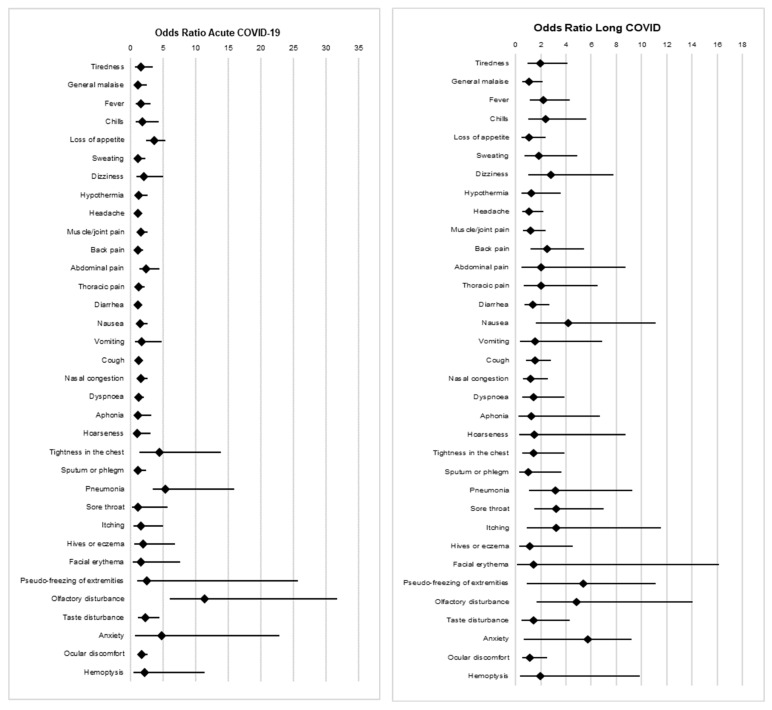
Signs and symptoms in patients with acute COVID-19 and long COVID: forest plot and logistic regression model.

Table 4 and Figure 1 present the signs and symptoms observed in patients with acute COVID-19 and long COVID, as determined by the binary logistic regression model. Significant findings were obtained for patients with acute COVID-19 who experienced an altered sense of smell (OR: 11.39; 95% CI: 5.98–31.74; *p* < 0.001), altered taste (OR: 2.28; 95% CI: 1.17–4.45; *p* = 0.016), chest tightness (OR: 4.37; 95% CI: 1.38–13.86; *p* = 0.012), pneumonia (*p* < 0.001), loss of appetite (*p* < 0.001), joint pain (*p* = 0.035), abdominal pain (*p* = 0.005), or eye discomfort (*p* = 0.009).

Similarly, significant findings were also obtained for patients who developed long COVID and presented with an altered sense of smell (OR: 4.83; 95% CI: 1.66–14.04; *p* = 0.004), pneumonia (OR: 3.18; 95% CI: 1.09–9.29; *p* = 0.035), fever (OR: 2.20; 95% CI: 1.14–4.27; *p* = 0.019), chills (OR: 2.38; 95% CI: 1.01–5.60; *p* = 0.047), sore throat (OR: 3.20; 95% CI: 1.47–6.99; *p* = 0.004), dizziness (OR: 2.80; 95% CI: 1.01–7.75; *p* = 0.048), nausea (OR: 4.20; 95% CI: 1.58–11.13; *p* = 0.004), or back pain (OR: 2.53; 95% CI: 1.182–5.43; *p* = 0.017).

The average number of pathologies reported by the patients was 0.80 ± 1.04 (95% CI: 0.74–0.86; range: 0–9), with significant differences observed among the groups (Kruskal–Wallis; *p* < 0.001). The group of patients with long COVID (1.40 ± 1.50; 95% CI: 1.17–1.63; range = 0–9) had a higher average number of pathologies compared to those with acute COVID-19 (0.82 ± 1.01; 95% CI: 0.70–0.93; range = 0–4) or without COVID-19 (0.71 ± 0.96; 95% CI: 0.66–0.77; range = 0–6). Table 5 presents the results for the prevalence of pathologies reported by patients, according to the comparison group. Among 20 pathologies studied, 11 were more frequently reported in patients with long COVID. Notably, overweight or obesity (18.3%; *p* < 0.001), asthma (9.4%; *p* = 0.007), hyperlipemia (8.4; *p* = 0.018), and anxiety syndrome (7.4%; *p* < 0.001) had a higher prevalence among patients with long COVID compared to those without.

Table 6 presents the results of the binary regression model analysing the pathologies in patients with acute COVID-19 and long COVID. Significant results were observed in patients with acute COVID-19, indicating a higher prevalence of the disease in people who were overweight or obese (higher prevalence of acute COVID-19 in individuals with overweight or obesity; OR: 1.48; 95% CI: 1.13–1.93; *p* = 0.004). In patients with long COVID, significant results were obtained for overweight or obesity (OR: 1.95; 95% CI: 1.39–2.74; *p* < 0.001), asthma (OR: 1.54; 95% CI: 1.01–2.39; *p* = 0.049), anxiety (OR: 3.15; 95% CI: 1.99–4.98; *p* < 0.001), and coagulation disorders (OR: 2.87; 95% CI: 1.95–3.84; *p* < 0.001), indicating a higher prevalence of long COVID in patients with these conditions.

## 4. Discussion

The objective of this study was to conduct a more comprehensive analysis of the clinical and epidemiological profiles of primary care professionals in Spain. Additionally, it was considered important to describe them in greater detail and compare them with the profiles of patients with acute COVID-19 infection and those who did not have it. On the other hand, it was also considered important to study the symptoms that turned out to be associated with a higher prevalence of being infected with COVID-19 or developing long COVID when experiencing them.

Our results indicated statistically significant differences in the presence of long COVID between men and women, with a higher prevalence among women. Women were also been shown to be more likely to develop the condition. The analysis of COVID-19 cases in healthcare workers reported to the Red Nacional de Vigilancia Epidemiológica (RENAVE) in 2020 confirmed a significantly higher prevalence of cases in women compared to men [32]. Findings from the literature also support the higher prevalence of long COVID among women [33,34,35,36]. This could be explained by the role of hormones in the acute phase of infection and even after recovery from the disease [33]. Additionally, it has been found that women exhibit a higher production of IgG antibodies, which may translate into a beneficial effect, leading to more favourable outcomes for women [5,37,38].

Likewise, statistically significant differences were observed in relation to the symptoms caused by COVID-19 infection. Symptoms were more pronounced in the group that had long COVID than in the sample that only had acute COVID-19, and even more pronounced than in those who were not infected. However, it is also noteworthy that the highest percentage of the population included in the study experienced symptoms resulting from COVID-19. For example, in the study by Carfi et al. [39], only 12.6% of patients were totally asymptomatic. The most common first symptom in both the acute COVID-19 and long COVID groups was tiredness or fatigue, followed by headache, muscle or joint pain, and dyspnoea. Similar results were reported in other studies, where 32% of the sample reported persistent fatigue [40], 87% fever and 91% malaise or general malaise [41]. It was also found that a large percentage of the population exhibited signs of dyspnoea, muscle weakness and headache, even weeks after testing negative for COVID-19 [13,42,43]. Similarly, a systematic review by Wong and Weitzer [44], which included 21 studies of COVID, suggests that fatigue was a predominant symptom in patients with long COVID in 12 of the studies.

Our results also demonstrated that certain symptoms can be associated with a higher prevalence of experiencing an acute COVID-19 infection or developing long COVID. Symptoms such as altered taste and smell, chest tightness, pneumonia, loss of appetite, joint pain, abdominal pain and/or eye discomfort were found to be associated with acute COVID-19. Similarly, in this population, there were symptoms associated with long COVID, including altered sense of smell, pneumonia, fever, chills, sore throat, dizziness, nausea, and/or back pain.

Other authors also emphasize the importance of establishing an association between symptoms and COVID-19 to facilitate the diagnosis of the disease [45]. Several studies have highlighted the high prevalence of patients experiencing alterations in taste and smell following the infection [46,47,48]. However, none of them analysed these as symptoms potentially associated with COVID-19 infection, nor did they examine the associations with the rest of the symptomatology. Regarding the symptoms associated with long COVID, Arjun et al. emphasized their importance in helping prioritize the at-risk population and designing new intervention plans, although contrary to our study, they found that gender was not associated with long COVID [49]. Similarly, there are no studies that examine the symptoms associated with long COVID. At this stage, it is important to highlight the multisystem effects of COVID-19, which can lead to complications when the person is infected or during the course of the disease. Although the epidemiology and pathophysiology of these symptoms are currently not well understood [10], the complications can affect the respiratory system [50] and the cardiovascular [51] renal [52], thromboembolic [53], neurological [54,55] and autoimmune systems [56]. The results of this study emphasized the presence of statistically significant differences in the average number of pathologies between people with long COVID and those who only had acute COVID or were COVID-negative; long COVID individuals showed a higher average number of pathologies, particularly overweight or obesity, asthma, hyperlipidaemia and anxiety syndrome. Similarly, both overweight and obesity have been shown to be associated with the increased prevalence of both COVID-19 infection and the development of long COVID, as well as asthma, anxiety and coagulation disorders. These results are consistent with other studies that suggest that people who are overweight or obese are more likely to have prolonged symptoms following COVID infection [57,58,59]. However, other studies claim that factors such as hypertension, chronic kidney disease, and immunocompromised states are related [60].

There was also a high prevalence of pathologies such as stroke, depression, coagulation disorders, renal failure and/or anaemia. Other studies also indicated an apparent association of COVID-19 with stroke, likely due to shared factors between them [55]. Similarly, regarding health complications and long-term effects of long COVID, mental health symptoms such as depression, anxiety or anxious syndrome, stress, psychiatric disorders, insomnia, etc., have been observed [61]. Likewise, Sonnweber et al. [62] demonstrated that disturbances in iron homeostasis and anaemia were common issues following COVID-19 infection and gradually decreased during their follow-up. A significant proportion of the population exhibited iron deficiency or persistent anaemia after COVID-19, which may contribute to a higher burden of persistent symptoms in these people. Although there are limited studies related on this topic, it has also been shown that persistent COVID can lead to loss of renal function months after infection (even in patients who did not require hospital admission). This represents an important clinical condition that should be given special attention in patients who have had COVID [63].

Our results also presented statistically significant data on the patients who had asthma when infected with the COVID-19 virus and subsequently developed persistent COVID. However, no significant data were obtained for people with chronic obstructive pulmonary disease (COPD), despite the fact that people who have asthma and/or COPD are at an increased risk of COVID-19 infection and developing more severe symptoms. It appears that these diseases are quite rare as comorbidities with COVID [64,65].

However, before concluding, it is worth mentioning the importance of these results for early diagnosis. The obtained data describe both the sociodemographic and clinical profiles of the healthcare population in Spain and their relationships with infection and the development of long COVID. This comes at a time when, despite the availability of reliable methods such as RT-PCR and serological tests as diagnostic tests, access to these tests is limited in other regions with low socio-economic levels, mostly developing countries. In addition, it is important to highlight the major health-related issues regarding the lack of services and shortages of ICU beds that have been and continue to be experienced in many areas [66,67].

This study had a number of limitations. The type of survey used (self-administered) can introduce an information selection bias.

In addition, the limited evidence on the clinical characteristics of COVID and long COVID in healthcare professionals made it difficult to compare with the results of our study. Similarly, the scarcity of studies analysing the associations of COVID-19 and long COVID symptoms made it difficult to compare our results, emphasizing the lack of studies examining associated symptoms of these conditions in healthcare professionals. Due to these biases, external validation of the study may be limited. However, it is important to consider that the results obtained in the study represent valuable data for the investigation of symptoms and/or complications associated with or derived from COVID-19 and long COVID in healthcare professionals. They hold great importance for future research on this disease, as well as for the design of studies and research protocols on persistent COVID. Additionally, a larger sample size of health professionals presenting COVID-19 and long COVID could address the differences between different professional categories.

Some strengths of this study include the significant number of participants who collaborated and the importance of the results associated with COVID-19 infection and long COVID development, given the limited existence of studies examining these aspects. It is of great significance to obtain a population profile and contribute to early diagnosis, as well as establish early treatment and intervention measures to prevent symptom persistence and/or severity.

## 5. Conclusions

In conclusion, our findings described the sociodemographic and clinical characteristics of healthcare workers of the SNH, who developed COVID-19, whether acute or long-term syndrome.

Thus, this study provided relevant information on the differences in symptomatology between healthcare workers who were infected with COVID-19, those who developed long COVID and those who were not infected. This information can be significant for the early detection of symptoms and for the diagnosis of the disease.

Regarding the previous pathologies of health workers, it is important to highlight the diseases that developed from the infection, such as nervous system disorders, and those previous pathologies that led to the development of long COVID. This will facilitate the implementation of multidisciplinary prevention and/or treatment programs aimed at improving the quality of life and well-being of this population group.

Finally, the highlights of this study include the following:

- The prevalence of long COVID was found to be higher in females, in addition to the greater probability of its development.
- The study revealed a high prevalence of fatigue in this population. However, there are other symptoms associated with long COVID, such as an altered sense of smell, pre-existing pneumonia and fever, sore throat, dizziness, nausea, and/or back pain.
- Certain symptoms associated with acute COVID-19 were also identified, such as an altered sense of taste and smell, chest tightness, and pneumonia.

Overweight or obese individuals are at a higher risk of contracting an acute COVID-19 infection or developing long COVID later on, as are those with asthma, anxiety, and coagulation disorders.

## Figures and Tables

**Table 1:** Sociodemographic characteristics according to comparison group.

Sociodemographic and Clinical Characteristics	Groups						Total n = 1490			*p*-Value
	Without COVID-19 n = 1087		Acute COVID-19 n = 251		Long COVID-19 n = 152					
	n	%	n	%	n	%	n	%	CI	
Sex										
Female	818	75.3	178	70.9	129	84.9	1125	75.5	73.3–77.7	0.007
Male	269	24.7	73	29.1	23	15.1	385	24.5	23.6–28.1	
Location										
Rural	0	0.0	2	0.8	26	17.3	28	1.9	1.2–2.6	<0.001
Urban	1087	100.0	249	99.2	126	82.3	1259	84.5	82.7–86.3	
Symptomatology										
Number of symptoms	2.64 ± 4.00		6.38 ± 5.26		14.60 ± 8.78			4.44 ± 6.05		

CI: Confidence Interval; SD: Standard Deviation.

**Table 2:** Sociodemographic characteristics of patients with acute COVID-19 and long COVID: logistic regression final models.

	Acute COVID-19					Long COVID				
	B Coefficient	OR	95% Confidence Interval		*p* Value	B Coefficient	OR	95% Confidence Interval		*p* Value
Sex (female vs. male)	0.07	1.08	0.852	1.37	0.521	0.64	1.90	1.32	2.74	<0.001
Location (rural vs. urban)	0.92	2.52	0.623	10.19	0.195	0.15	1.16	0.80	1.70	0.418

OR: Odds Ratio.

**Table 3:** Symptoms, signs reported by patients according to comparison group.

Symptoms, Signs and Clinical Pictures	Groups						Total n = 1490		*p*-Value
	Without COVID-19 n= 1087		Acute COVID-19 n = 251		Long COVID n = 152				
	n	%	n	%	n	%	n	%	
General or Nonspecific Symptoms									
Tiredness	147	13.5	125	49.8	120	78.9	392	26.3	<0.001
General malaise	141	13.0	105	41.8	87	57.2	333	22.3	<0.001
Fever	90	8.7	84	33.5	85	55.9	259	17.4	<0.001
Chills	72	6.6	37	14.7	66	43.4	175	11.7	<0.001
Loss of appetite	53	4.9	56	22.3	57	37.5	166	11.1	<0.001
Sweating	32	2.9	24	9.6	55	36.2	111	7.4	<0.001
Dizziness	25	2.5	11	4.9	68	41.6	104	6.6	<0.001
Hypothermia	34	3.1	19	7.6	41	27.0	94	6.3	<0.001
Body Aches									
Headache	242	22.3	115	45.8	106	69.7	463	31.1	<0.001
Muscle/joint pain	133	12.2	101	40.2	96	63.2	330	22.1	<0.001
Sore throat	194	17.8	71	30.3	3	9.1	268	19.8	0.006
Back pain	60	5.8	38	15.1	71	46.7	172	11.5	<0.001
Abdominal pain	27	2.5	26	10.4	52	34.2	105	7.0	<0.001
Thoracic pain	32	2.9	16	6.4	48	31.6	96	6.4	<0.001
Stomach pain	29	2.7	14	5.6	50	32.9	93	6.2	<0.001
Ear pain	3	0.3	0	0.0	0	0.0	3	0.3	0.872
Digestive									
Diarrhoea	93	8.6	43	17.1	66	43.4	202	13.6	<0.001
Nausea	22	2.0	12	4.8	46	30.3	80	5.4	<0.001
Vomiting	16	1.5	5	3.7	31	20.4	52	3.5	<0.001
Respiratory									
Cough	201	18.5	105	41.8	87	57.2	393	26.4	<0.001
Nasal congestion	117	10.8	56	22.3	60	39.5	233	15.6	<0.001
Dyspnoea	48	4.4	39	15.5	93	61.2	180	12.1	<0.001
Aphonia	48	4.4	27	10.8	52	34.2	127	8.5	<0.001
Hoarseness	46	4.2	23	9.2	52	34.2	121	8.1	<0.001
Tightness in the chest	33	3.0	16	6.4	59	38.8	108	7.2	<0.001
Sputum or phlegm	21	1.9	11	4.4	44	28.9	76	5.1	<0.001
Pneumonia	10	0.9	16	6.4	40	26.3	66	4.4	<0.001
Rhinitis	0	0.0	6	2.4	53	34.9	59	4.0	<0.001
Sore throat	3	0.3	2	0.9	0	0.0	5	0.4	0.522
Dermatological									
Itching	13	1.2	7	2.8	33	21.7	53	3.6	<0.001
Hives or eczema	17	1.6	6	2.4	24	15.8	47	3.2	<0.001
Facial erythema	12	1.1	3	1.2	30	19.7	45	3.0	<0.001
Pseudo-freezing of extremities	3	0.3	8	3.2	34	22.3	45	3.0	<0.001
Inflammation of the fingers	2	0.2	2	0.8	12	7.9	16	1.1	<0.001
Livedo reticularis	3	0.3	1	0.4	0	0.0	4	0.3	0.481
Coldness in the extremities	0	0.0	1	0.4	0	0.0	1	0.1	0.804
Neurological and Psychological									
Olfactory disturbance	32	2.9	104	41.4	88	57.9	224	15.0	<0.001
Taste disturbance	32	2.9	89	35.5	77	50.7	198	13.3	<0.001
Anxiety	2	0.2	5	2.0	34	22.4	41	2.8	<0.001
Polyneuropathy	1	0.1	1	0.4	0	0.0	2	0.1	0.492
Sensory disturbance	1	0.1	0	0.0	0	0.0	1	0.1	0.882
Insomnia	0	0.0	0	0.0	0	0.0	0	0.0	------
Others									
Ocular discomfort	66	6.0	46	18.3	45	29.6	156	10.5	<0.001
Haemoptysis	2	0.2	1	0.4	10	6.6	13	0.9	<0.001
Cold sores	1	0.1	0	0.0	0	0.0	1	0.1	0.874

**Table 4:** Signs and symptoms in patients with acute COVID-19 and long COVID: logistic regression model.

	Acute COVID-19				Long COVID			
	OR	95% Confidence Interval		*p* Value	OR	95% Confidence Interval		*p* Value
Tiredness	1.54	0.70	3.37	0.285	1.96	0.93	4.12	0.078
General malaise	1.17	0.56	2.46	0.670	1.05	0.51	2.15	0.893
Fever	1.54	0.76	3.10	0.228	2.2	1.14	4.27	0.019
Chills	1.82	0.77	4.31	0.175	2.38	1.01	5.60	0.047
Loss of appetite	3.61	2.42	5.39	<0.001	1.06	0.47	2.39	0.878
Sweating	1.16	0.60	2.21	0.664	1.82	0.68	4.91	0.234
Dizziness	2.08	0.86	4.98	0.102	2.8	1.01	7.75	0.048
Hypothermia	1.28	0.64	2.58	0.486	1.27	0.45	3.58	0.655
Headache	1.12	0.73	1.71	0.609	1.08	0.53	2.2	0.844
Muscle/joint pain	1.62	1.03	2.55	0.035	1.16	0.56	2.38	0.695
Back pain	1.11	0.63	1.94	0.721	2.53	1.18	5.43	0.017
Abdominal pain	2.40	1.31	4.41	0.005	2.05	0.49	8.71	0.329
Thoracic pain	1.23	0.69	2.18	0.485	2	0.62	6.49	0.248
Diarrhoea	1.11	0.70	1.77	0.659	1.38	0.70	2.71	0.355
Nausea	1.41	0.75	2.64	0.290	4.2	1.58	11.13	0.004
Vomiting	1.74	0.63	4.77	0.284	1.57	0.36	6.86	0.552
Cough	1.26	0.86	1.86	0.241	1.52	0.83	2.81	0.421
Nasal congestion	1.60	0.99	2.57	0.055	1.21	0.57	2.55	0.616
Dyspnoea	1.20	0.73	1.98	0.465	1.41	0.51	3.89	0.431
Aphonia	1.15	0.41	3.22	0.793	1.24	0.23	6.66	0.804
Hoarseness	1.04	0.36	3.01	0.936	1.51	0.26	8.75	0.649
Tightness in the chest	4.37	1.38	13.86	0.012	1.41	0.51	3.89	0.512
Sputum or phlegm	1.10	0.51	2.37	0.802	0.98	0.27	3.61	0.976
Pneumonia	5.36	3.40	15.93	<0.001	3.18	1.09	9.29	0.035
Sore throat	1.07	0.20	5.67	0.067	3.2	1.47	6.99	0.004
Itching	1.52	0.47	4.95	0.483	3.24	0.91	11.56	0.070
Hives or eczema	1.88	0.52	6.84	0.339	1.1	0.27	4.55	0.892
Facial erythema	1.55	0.32	7.61	0.589	1.45	0.13	16.13	0.763
Pseudo-freezing of extremities	2.51	0.97	25.68	0.054	5.4	0.91	11.09	0.064
Olfactory disturbance	11.39	5.98	31.74	<0.001	4.83	1.66	14.04	0.004
Taste disturbance	2.28	1.17	4.45	0.016	1.4	0.46	4.28	0.550
Anxiety	4.78	0.64	22.89	0.128	5.72	0.65	9.22	0.106
Ocular discomfort	1.72	1.15	2.58	0.009	1.13	0.50	2.52	0.770
Haemoptysis	2.13	0.40	11.41	0.378	1.96	0.32	9.85	0.280

OR: Odds Ratio.

**Table 5:** Pathologies reported by patients according to comparison group.

Diseases	Groups						Total n = 1490		*p*-Value
	Without COVID19 n = 1087		Acute COVID19 n = 251		Long COVID n = 152				
	n	%	n	%	n	%	n	%	
Overweight or obese	173	15.9	51	20.3	49	32.2	273	18.3	<0.001
Arterial hypertension	94	8.6	30	12.0	16	10.5	140	9.4	0.154
Asthma	92	8.5	23	9.2	25	16.4	140	9.4	0.007
Hyperlipidaemia	86	7.9	20	8.0	19	12.5	125	8.4	0.018
Anxiety syndrome	61	5.6	15	6.0	34	22.4	110	7.4	<0.001
Endocrine disease	62	5.7	22	8.8	8	5.3	92	6.2	0.137
Autoimmune disease	44	4.0	6	2.4	5	3.3	55	3.7	0.534
Depression	25	2.3	4	1.6	13	8.6	42	2.8	<0.001
Diabetes Mellitus	21	1.9	10	4.0	1	0.7	32	2.1	0.015
Heart disease	18	1.7	7	2.8	3	2.0	28	1.9	0.436
Cancer	14	4.0	3	1.2	1	0.7	18	1.2	0.770
Coagulation disorders	2	0.2	1	0.4	13	8.6	16	1.1	<0.001
Immunosuppression	11	1.0	2	0.8	4	2.6	17	1.1	0.016
Allergic rhinitis	8	0.7	1	0.4	0	0.0	9	0.7	0.768
COPD	8	0.7	2	0.8	1	0.7	11	0.7	0.963
Kidney disease	5	0.5	0	0.0	5	3.3	10	0.7	<0.001
Stroke/thrombosis	1	0.1	2	0.8	6	3.9	9	0.6	<0.001
Sleep apnoea syndrome	5	0.5	0	0.0	0	0.0	5	0.4	0.531
Anaemia	0	0.0	1	0.4	1	3.2	2	0.1	0.001
Fibromyalgia	1	0.1	0	0.0	0	0.0	1	0.1	0.882

**Table 6:** Clinical pathologies of patients with acute COVID-19 and long COVID: logistic regression final models.

	Acute COVID-19					Long COVID				
	B Coefficient	OR	95% Confidence Interval		*p* Value	B Coefficient	OR	95% Confidence Interval		*p* Value
Arterial hypertension	0.15	1.16	0.83	1.64	0.374	0.15	1.16	0.71	1.89	0.542
DM	0.49	1.64	0.86	3.11	0.129	-	-	-	-	-
Hyperlipidaemia	0.22	1.24	0.86	1.79	0.233	0.22	1.25	0.78	2.00	0.353
Overweight or obese	0.39	1.48	1.13	1.93	0.004	0.67	1.95	1.39	2.74	<0.001
Asthma	0.14	1.15	0.81	1.64	0.762	0.41	1.54	1.01	2.39	0.049
Anxiety syndrome	0.70	2.03	1.35	3.05	<0.001	1.14	3.15	1.99	4.98	<0.001
Depression	0.32	1.37	0.70	2.70	0.354	0.04	1.04	0.48	2.24	0.917
Coagulation disorders	0.44	1.55	0.13	17.87	<0.001	3.35	2.87	1.95	3.84	<0.001

OR: Odds Ratio; B Coefficient: Beta Coefficient.
